# Ten years to VISION 2020: why information matters

**Published:** 2010-12

**Authors:** Peter Ackland

**Affiliations:** Chief Executive, International Agency for the Prevention of Blindness (IAPB), London School of Hygiene and Tropical Medicine, Keppel Street, London WC1E 7HT, UK.

**Figure:**
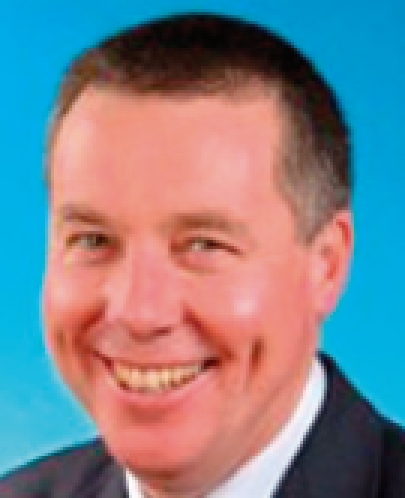


Why do we collect information in our eye care programmes? One important reason is that we use information to enhance our knowledge and then apply that knowledge to improve what we do.

Information is the first step in a process of learning and improvement that enables us to make better decisions, improve the systems and places we work in, and enhance the quality of our own individual work.

Information can help us improve; the trick is that we only collect the information we really need. We should neither overburden ourselves with more information than we can use, nor should we overload our colleagues with unnecessary requests for information when they should rather be delivering eye care to patients.

We need the buy-in and cooperation of our colleagues if we want to collect accurate information; this means that everyone has to be motivated to make it work. Some time ago, when designing a reporting format for VISION 2020 programmes, a wise colleague advised me that, unless the person who was collecting information could see the benefit to them of doing this, it was just a waste of everyone's time.

So why do we all need to invest some of our precious time in collecting and providing good information? What are the benefits to **you**?

## Quality assurance and improvement

Just about everybody I have ever met in the health profession wants to do a good job for the patients they are serving; it is part of their motivation for being a health professional. Everyone has a responsibility to do the best they possibly can in the environment they work in and to try and get better at what they do. Improvement comes from experience, learning from others, and keeping abreast of the latest developments. It also involves looking at how, as an individual, one is presently performing, being self-critical, and trying to improve. In the article on clinical auditing to improve patient outcomes, David Yorston and Richard Wormald outline just how important it is to monitor the outcome of surgery and compare performance over a period of time (page 48). Auditing is not about policing and pointing the finger when things go wrong-it is about trying to learn from what one is currently doing and, where appropriate, improve it. Recently,^1^ it was reported that, in eight recent population-based surveys in various African countries, normal vision (visual acuity of ≥ 6/18 or better) had only been restored to between 23% and 59% of eyes operated on to remove cataracts. There are many reasons for this, which we do not have the space to explore here, but can anyone reasonably argue against the need for self-auditing and self-improvement in the light of such findings? Yes, it takes time to capture and analyse the data, but if it helps even a handful of one's patients see better, then surely it is time well spent.

**Figure:**
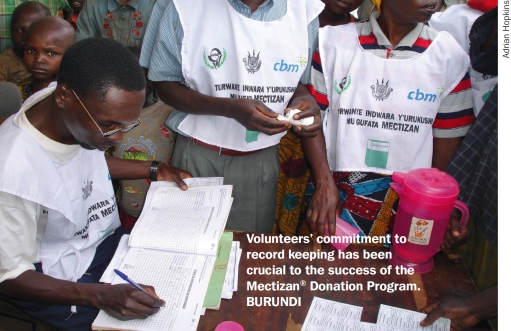
Volunteers' commitment to record keeping has been crucial to the success of the Mectizan® Donation Program. BURUNDI

Adrian Hopkins

But it is not just the surgeons who are responsible for quality improvement; everyone in the eye care team has an important role to play. The article by Sue Stevens and Dianne Pickering (page 44) gives some really practical advice on how nurses can help improve the quality of care received by patients through the keeping of good nursing records. Ingrid Mason and Jonathan Pons give ideas and practical examples of how patient records can be managed at the eye care facility level, which requires the cooperation and dedication of management, doctors, nurses, and administrative staff (page 46).

At the community level, I have always marvelled at how one can visit some of the remotest villages in rural Africa and ask one of the community distributors - a volunteer from the village-for his/her records of the persons he/she has distributed Mectizan® to and how many tablets were given to each. Invariably, the distributor is able to show one the most pristine and complete records and can proudly explain, in great detail, what has been done. Equally impressively, well-attended community meetings are held to discuss the performance of the local community distributors using the collected data. This in turn gets summated to give national and global data. In the end, the work of some 250,000 distributors, working in 30 countries, results in 75 million people a year being treated. As Adrian Hopkins shows (page 53), the motivation of the distributors and their whole-hearted commitment to record keeping has been crucial to the success of onchocerciasis control programmes. As a result, the elimination of this blinding disease is a real possibility in many areas.

In Pakistan, considerable investment has been made in training Lady Health Workers to treat very basic eye problems and refer patients with more severe conditions. Recently, a new management information service was developed that enabled and required the Lady Health Workers to report the number of people they treated and referred with eye problems. This has been implemented with extraordinary success: policy makers now understand just how many people suffer from eye conditions in the community. It has also been a huge morale boost to the Lady Health Workers themselves as they see the value of their work being recognised and acted upon.

How come, then, do many of us well-trained professionals find it so hard to complete data records, whilst village volunteers in Africa and Asia are so successful? The answer, I think, is that data requested of the volunteers are confined to only what it is absolutely essential to know. As my wise colleague pointed out, the volunteers who keep the records clearly see the benefit to them of keeping those records; they understand the need for these data and how they are used to better understand the eye care situation. The volunteers also receive feedback on their own performance, which assures them that someone has actually done something with the data they produce. Most take pride in collecting data, which meets the simple human need to feel good about one's own contribution to the world and to be able to say: “Here is proof of what I have done.”

## Advocacy

Advocacy is about using evidence to argue for a particular change one believes in and wants to make happen. We all advocate for things we want and certainly the availability of good information is a powerful tool when presenting a case.

Many (if not everyone) reading this article will be working in an environment with too few resources. Many of you will not have the drugs, consumables, or equipment to do the job you need to do. Your hospital administrators will sympathise but explain that, with so many other demands, their hands are tied. Keeping good records of your work will help you to argue your case in the face of limited resources and competing priorities. Data alone will not guarantee that you get your desired resources, but it will certainly make your argument stronger.

Launched in 1999, VISION 2020 is roughly halfway through and we have ten years left to achieve our goal of the elimination of avoidable blindness. We have achieved much: more than 100 countries have a national plan to tackle avoidable blindness and visual impairment, there is a World Health Assembly-adopted Action Plan and there are now many tried and tested programme approaches. But our biggest challenge today is to implement all these plans and scale them up from project level to full country-wide programmes to ensure that high-quality and equitable eye health services are available to all (page 55). Good data will be essential as we advocate for the resources to make this happen.
